# Supplementary material for: Graph neural networks learn emergent tissue properties from spatial molecular profiles
Source: Nat Commun. 2025 Sep 25;16:8419. doi: 10.1038/s41467-025-63758-8 (PMC12462520; doi:10.1038/s41467-025-63758-8)
Supplement: Supplementary file 1 — Supplementary Information [file 41467_2025_63758_MOESM1_ESM.pdf]

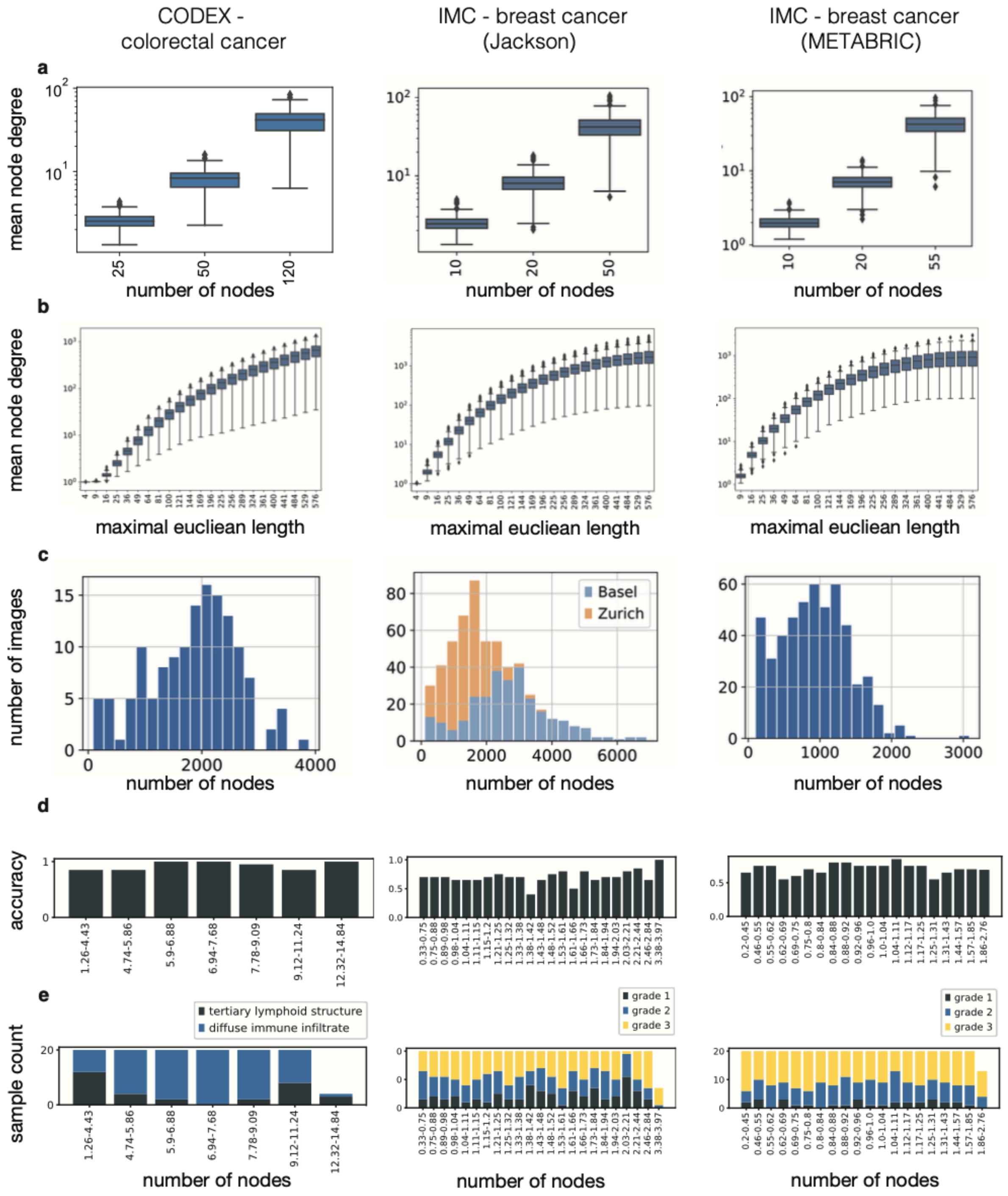

Supp. Figure 1: Graph summary statistics of analyzed data. Mean node-degree per image by radius length used in benchmarks **(a)** and in a scan across **(b)**, number of nodes per image **(c)**, binned accuracy by number of nodes in graph **(d)** and label distribution over graph size bins **(e)** for CODEX - colorectal cancer (left), IMC - breast cancer (Jackson) (middle) and IMC - breast cancer (METABRIC) (right) datasets.

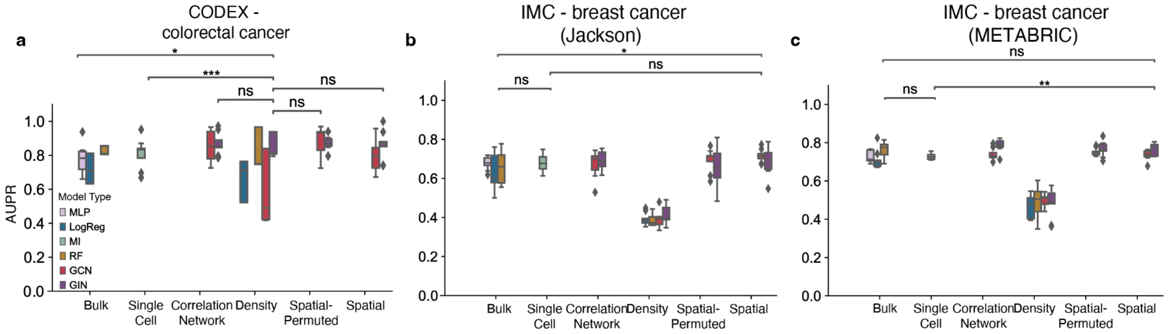

Supp. Figure 2: Baseline models for graph neural networks on molecular feature space. (a-c) Three separate applications of graph neural networks to predict tumor phenotypes on the IMC - breast cancer (Jackson), IMC - breast cancer (METABRIC) and anatomical label on the CODEX - colorectal cancer datasets. The neural networks are a multi-layer perceptron in Bulk, a multi-instance (MI) in Single Cell, and a GCN and GIN in the Graph scenario GNN. Baseline models are random forests (RF) and regression models (Logistic regression) trained on the mean feature value across the graph (Bulk), the node degree distribution per graph (Density), or the correlation network of k-nearest neighbor (KNN) graphs based on gene expression similarities (Methods). Statistical significance was assessed using a two-sided unpaired Student's t-test ($p > 0.05$, ns; $p < 0.05$, *; $p < 0.01$, **; $p < 0.001$, ***). Box plots show the median (center line), the 25th and 75th percentiles (bounds of box), and whiskers extending to the most extreme data points within 1.5× the interquartile range; outliers are shown as individual points.

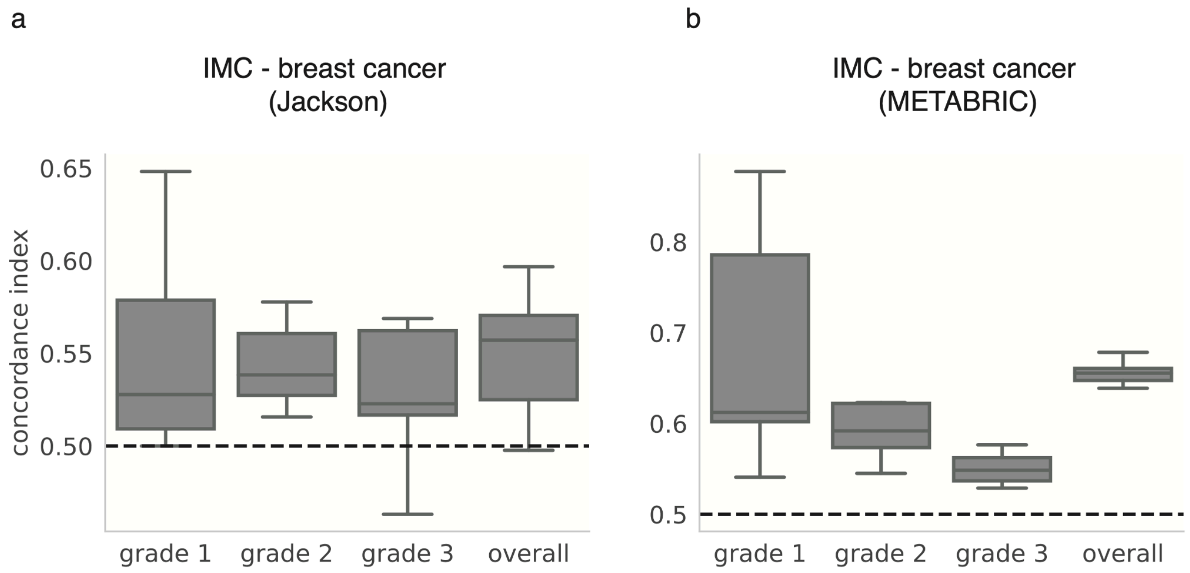

Supp. Figure 3: Patient survival analysis based on graph embeddings. Boxplots showing the censored concordance index values computed based on the first principal component of the graph embeddings for all 9 cross-validation splits, for breast cancer datasets, **(a)** IMC - Jackson and **(b)** IMC - METABRIC. Box plots show the median (center line), the 25th and 75th percentiles (bounds of box), and whiskers extending to the most extreme data points within 1.5× the interquartile range; outliers are shown as individual points.

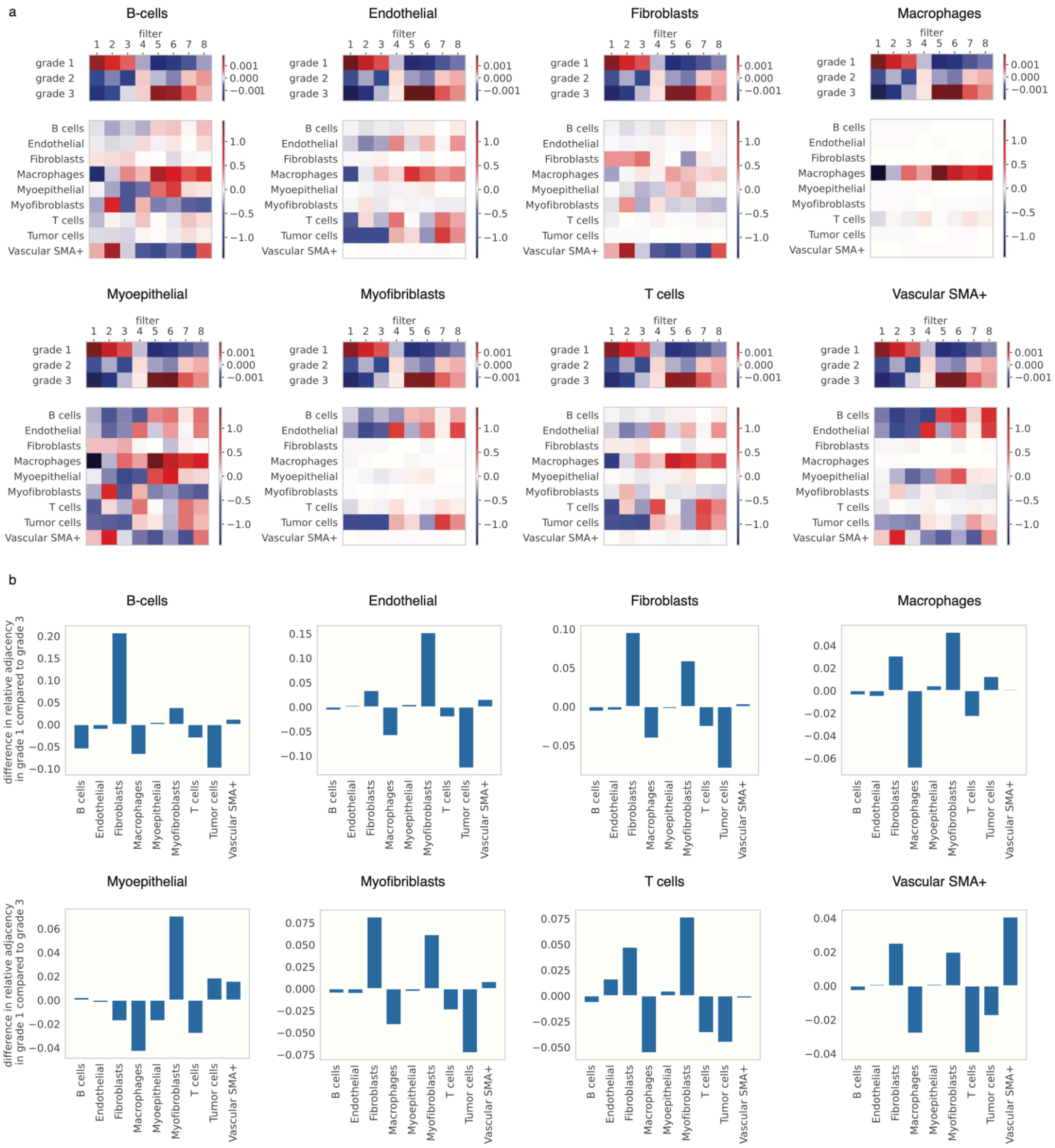

Supp. Figure 4: Attention filters from graph attention (GAT) model. The analyses presented concern the IMC - breast cancer (METABRIC) dataset. **(a)** Attention filters from the GAT model for the eight different cell types across the cancer grades. **(b)** Bar plots of the differences in relative adjacency for the different cell type neighbors in grade 1 compared to grade 3.

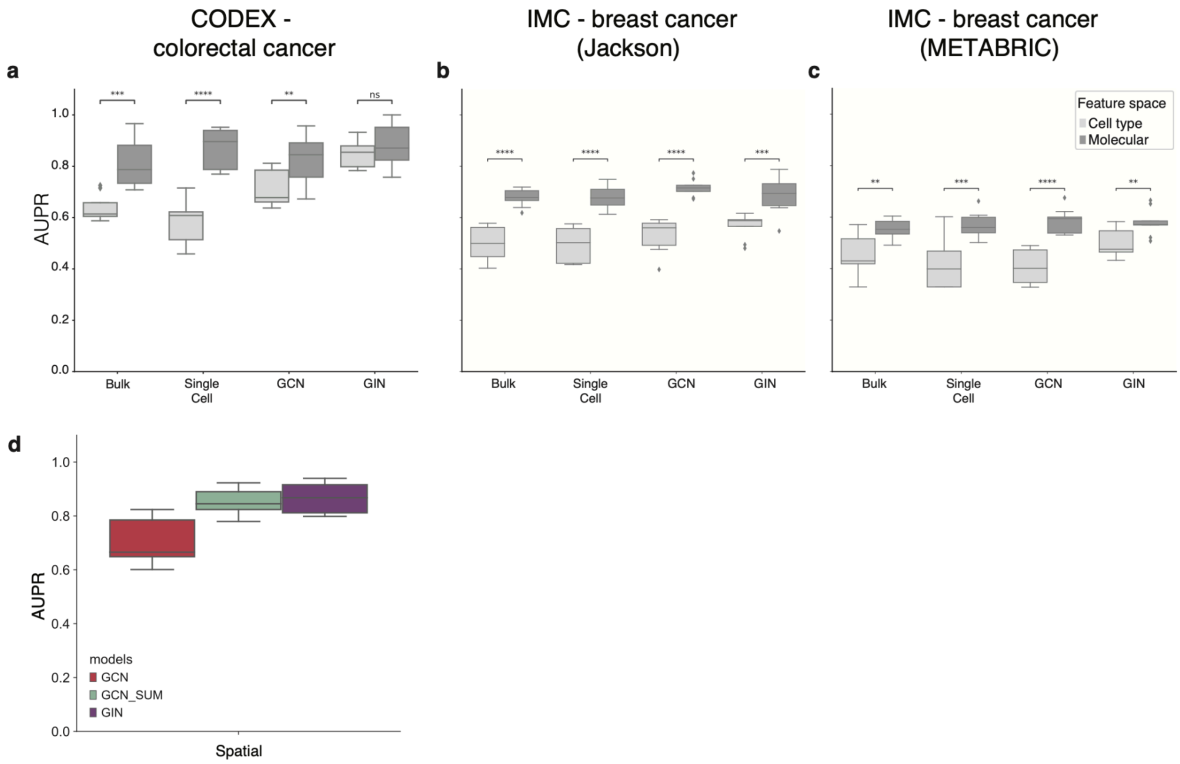

Supp. Figure 5: Training of models that predict anatomical labels and cancer grade based on cell type and molecular feature space. (a-c) Comparison between the performance of the molecular and binary cell types features for the different datasets: (a) CODEX - colorectal cancer, (b) IMC - breast cancer (Jackson), and (c) IMC - breast cancer (METABRIC.) **(d)** Comparison of different aggregation functions for graph models on colorectal cancer dataset. GCN with default aggregation function (GCN), GCN with sum aggregation function (GCN_SUM) and GIN with default aggregation function (GIN). Statistical significance was assessed using a two-sided unpaired Student's t-test ($p > 0.05$, ns; $p < 0.05$, *; $p < 0.01$, **; $p < 0.001$, ***). Box plots show the median (center line), the 25th and 75th percentiles (bounds of box), and whiskers extending to the most extreme data points within 1.5× the interquartile range; outliers are shown as individual points.

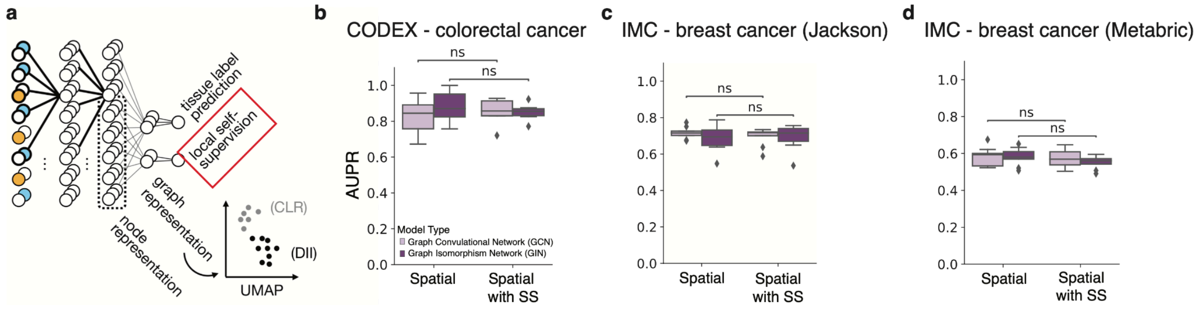

Supp. Figure 6: Graph models with self-supervision. (a) The spatial context of each cell can be formally represented by a graph in which edges are weighted based on the distance between nodes. Each sample can be represented as one such graph, where nodes are colored by the measured cell features. We perform prediction with a model that consists of graph neural network layers to produce node embeddings, followed by pooling over nodes and a final classification network. In addition, the node embeddings of connected components of nodes on the spatial proximity graph can be aggregated for local self-supervision tasks, such as reconstruction of adjacent clusters' cell type composition. *dotted line*: connected component of nodes on spatial proximity graph. **(b-d)** Comparison of performance of graph models with and without self-supervision task on the different datasets: (b) CODEX - colorectal cancer, (c) IMC - breast cancer (Jackson), and (d) IMC - breast cancer (METABRIC) trained on molecular features using different measures (N=9 nested cross validations per method and dataset).

Statistical significance was assessed using a two-sided unpaired Student's t-test ($p > 0.05$, ns; $p < 0.05$, *; $p < 0.01$, **; $p < 0.001$, ***). Box plots show the median (center line), the 25th and 75th percentiles (bounds of box), and whiskers extending to the most extreme data points within 1.5× the interquartile range; outliers are shown as individual points.

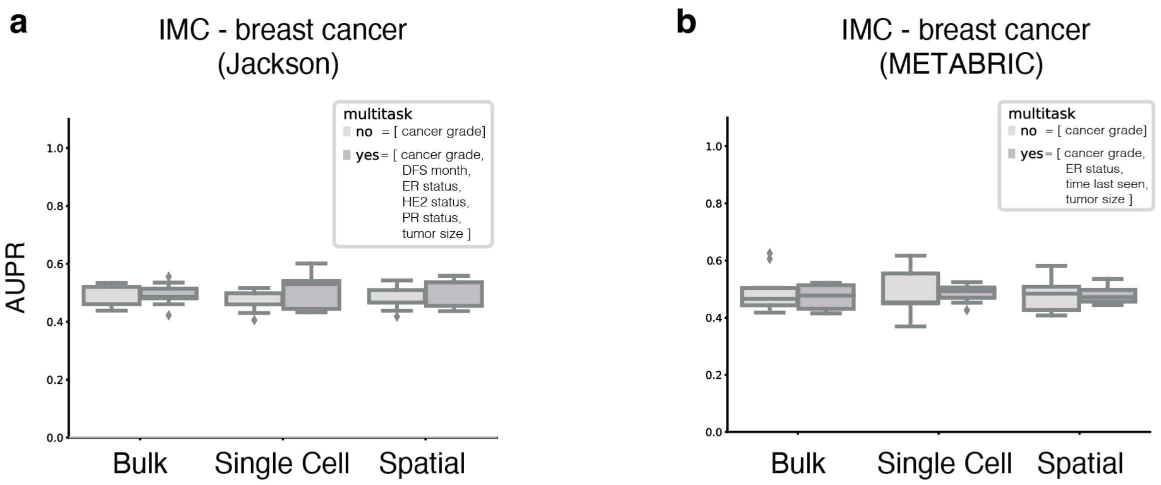

Supp. Figure 7: Multitask learning models. On breast cancer datasets, **(a, b)** shown is the accuracy on test set based on number of tasks (upper panel) and epoch-wise training of multi-tasking models (lower panel) on **(a)** IMC - breast cancer (Jackson) dataset, and **(b)** IMC - breast cancer (METABRIC). *target* with only the cancer grade and *multitask* with cancer grade, disease-free survival (DFS) month, estrogen receptor (ER) status HER2 status, progesterone receptor (PR) status and tumor size. Box plots show the median (center line), the 25th and 75th percentiles (bounds of box), and whiskers extending to the most extreme data points within 1.5× the interquartile range; outliers are shown as individual points.
